# Loss of DRO1/CCDC80 in the tumor microenvironment promotes carcinogenesis

**DOI:** 10.18632/oncotarget.28084

**Published:** 2022-04-11

**Authors:** Jessica I. Christian, Agnieszka Pastula, Andreas Herbst, Jens Neumann, Maximilian K. Marschall, Andrea Ofner, Heike Zierahn, Marlon R. Schneider, Eckhard Wolf, Michael Quante, Frank T. Kolligs

**Affiliations:** ^1^Institute of Molecular Animal Breeding and Biotechnology, Gene Center, Ludwig Maximilian University of Munich, Munich 81377, Germany; ^2^Gastroenterologie II, Klinikum rechts der Isar, Technical University of Munich, Munich 81675, Germany; ^3^Department of Medicine II, Ludwig Maximilian University of Munich, Munich 81377, Germany; ^4^Institute of Laboratory Medicine, University Hospital, Ludwig Maximilian University of Munich, Munich 81377, Germany; ^5^Institute of Pathology, Ludwig Maximilian University of Munich, Munich 80337, Germany; ^6^German Cancer Consortium (DKTK), Heidelberg 69120, Germany; ^7^German Cancer Research Center (DKFZ), Heidelberg 69120, Germany; ^8^Department of Internal Medicine and Gastroenterology, HELIOS Klinikum Berlin-Buch, Berlin 13125, Germany; ^*^These authors contributed equally to this work

**Keywords:** DRO1, CCDC80, colorectal cancer, tumor microenvironment, tumor suppressor

## Abstract

Tumors are composed of the tumor cells and the surrounding microenvironment. Both are closely interwoven and interact by a complex and multifaceted cross-talk which plays an integral part in tumor initiation, growth, and progression. *Dro1/Ccdc80* has been shown to be a potent suppressor of colorectal cancer and ubiquitous inactivation of *Dro1/Ccdc80* strongly promoted colorectal carcinogenesis in *Apc^Min/+^* mice and in a chemically-induced colorectal cancer model.

The aim of the present study was to investigate whether *Dro1/Ccdc80*’s tumor suppressive function is tumor-cell-autonomous. Expression of *Dro1/Ccdc80* in cancer cells had no effect on both colon tumor development in *Apc^Min/+^* mice and formation of xenograft tumors. In contrast, DRO1/CCDC80 loss in the microenvironment strongly increased tumor growth in xenograft models, inhibited cancer cell apoptosis, and promoted intestinal epithelial cell migration. Moreover, stromal *Dro1/Ccdc80* inactivation facilitated formation of intestinal epithelial organoids. Expression analyses showed *Dro1/Ccdc80* to be significantly down-regulated in murine gastric cancer associated fibroblasts, in *Apc^Min/+^* colon tumor primary stromal cells and in microdissected stroma from human colorectal cancer compared to normal, non-tumor stroma. Our results demonstrate epithelial derived DRO1/CCDC80 to be dispensable for intestinal tissue homeostasis and identify Dro1/Ccdc80 as tumor suppressor in the tumor microenvironment.

## INTRODUCTION

*Dro1/Ccdc80* has been identified as a tumor suppressor of colorectal, thyroid, and ovarian cancer [1–3]. *Dro1/Ccdc80* suppresses anchorage independent growth [2], inhibits migration of cancer cells [4] and induces sensitization to detachment-induced apoptosis [2]. Down-regulation of *Dro1/Ccdc80* has been shown in primary human colorectal, thyroid, and melanoma skin cancer [1, 2, 4, 5]. In *Apc^Min/+^* mice ubiquitous inactivation of *Dro1/Ccdc80* results in early death, a significant increase in the colonic tumor load, and the regular formation of adenocarcinoma in the colon [1]. Loss of DRO1/CCDC80 increases multiplicity of preneoplastic aberrant crypt foci and colonic tumors in carcinogen-induced colon carcinogenesis and promotes formation of colon adenocarcinoma during inflammation-driven carcinogenesis [6]. In colon tumors from *Apc^Min/+^* mice loss of DRO1/CCDC80 induces ERK1/2 phosphorylation and leads to c-MYC oncogene activation [1]. The *Dro1/Ccdc80* tumor suppressor function has been reviewed in detail in [7].

Tumors are composed of two distinct components, the tumor cells themselves and the surrounding microenvironment, also called the tumor stroma. The tumor stroma comprises capillaries, activated fibroblasts, nerves, basement membrane, extracellular matrix, and immune cells [8, 9]. Epithelial tumor cells and stromal cells, both closely interwoven within the tumor, interact by a complex and multifaceted paracrine cross-talk, which plays an integral part in tumor initiation, growth, and progression [8–11]. The stromal compartment is known to stimulate tumor cell proliferation and mediate evasion of tumor cells from apoptosis, promote continuous angiogenesis, and contribute to the invasive and metastatic process [11, 12]. Moreover, tumor stromal cells convey drug sensitivity as well as therapeutic resistance [13] and provide prognostic and response-predictive information [14].

To date, the tumor suppressor role of *Dro1/Ccdc80* *in vivo* has always been addressed by ubiquitous gene inactivation in mice [1, 3, 6]. For the study of tumorigenesis this approach implicates *Dro1/Ccdc80* deficiency in both the tumor parenchyma and the tumor microenvironment. The aim of the present study was to investigate whether *Dro1/Ccdc80*’s tumor suppressive function is tumor-cell-autonomous.

## RESULTS

### Colon tumor development is unaffected by epithelial *Dro1/Ccdc80*

**Figure 1:**
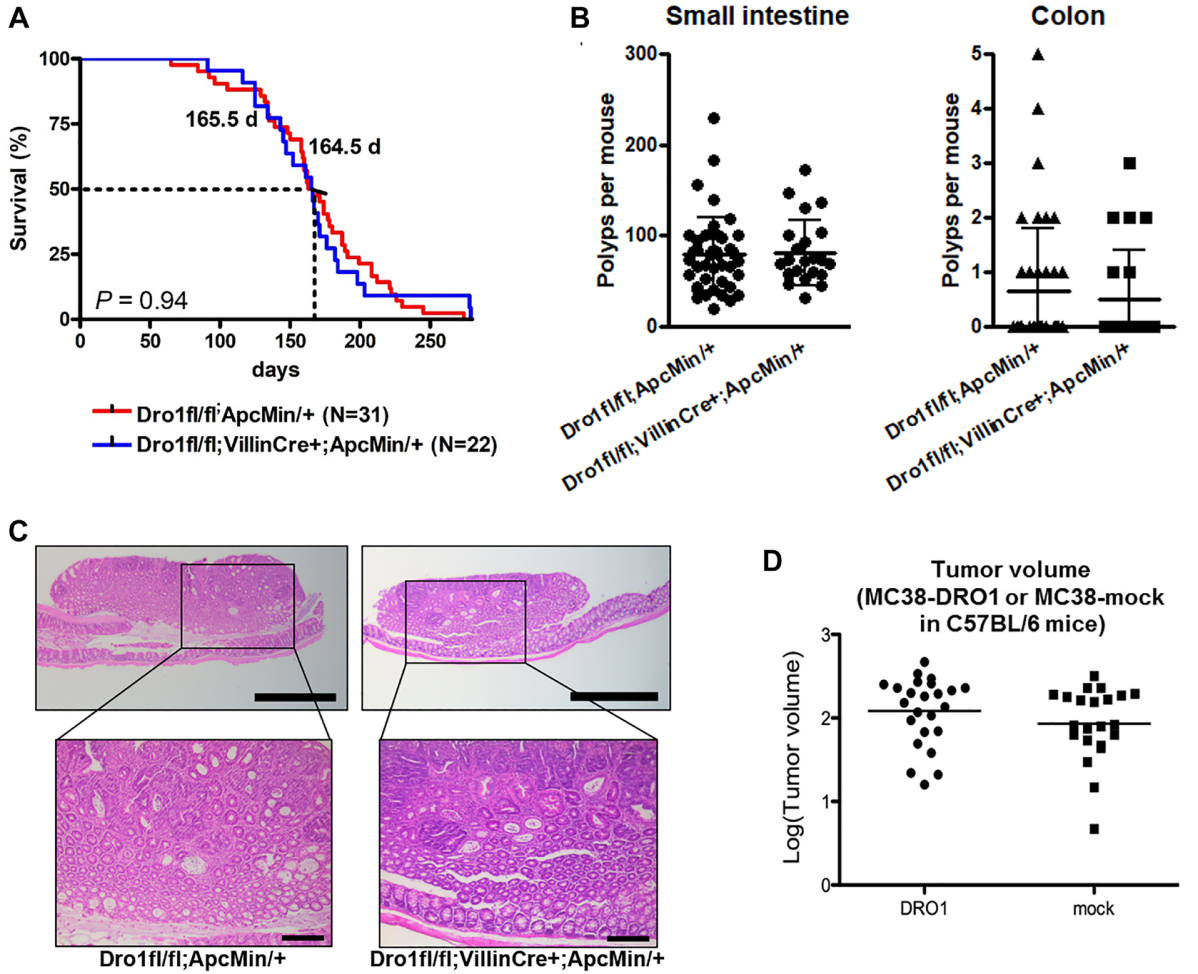
Colon tumor development is unaffected by epithelial Dro1/Ccdc80. (**A**) Survival of *Dro1^−/−^;VillinCre^+^;Apc^Min/+^* (*n* = 22) and *Dro1^fl/fl^;Apc^Min/+^* control (*n* = 31) mice. (**B**) Number of polyps per mouse in the small intestine and colon of moribund *Dro1^−/−^;VillinCre^+^;Apc^Min/+^* (*n* = 22) and *Dro1^fl/fl^;Apc^Min/+^* control (*n* = 42) mice. Error bars represent standard deviations. (**C**) Representative pictures of colon tumors from *Dro1^−/−^;pVillinCre^+^;Apc^Min/+^* and *Apc^Min/+^* control mice. H&E-staining. Scale bars, 500 μm and 200 μm. (**D**) Tumor volume of MC38-DRO1 and MC38-mock colorectal cancer cells subcutaneously injected into C57BL/6 mice (*n* = 24 tumors for MC38-DRO1 and *n* = 21 tumors for MC38-mock).

Ubiquitous inactivation of *Dro1/Ccdc80* in *Apc^Min/+^* mice results in early death, a dramatic increase in colon tumor number, and formation of adenocarcinoma in the colon [1]. To investigate whether tumor suppression by *Dro1/Ccdc80* is tumor-cell-autonomous, we inactivated *Dro1/Ccdc80* specifically in the intestinal epithelium of *Apc^Min/+^* mice. Therefore, *Apc^Min/+^* mice carrying floxed *Dro1/Ccdc80* alleles were crossed to mice expressing *Cre* recombinase under control of the intestinal epithelium specific *Villin* promoter [15] to generate *Dro1^fl/fl^;VillinCre^+^;Apc^Min/+^* mice and *Dro1^fl/fl^;Apc^Min/+^* control littermates. Correct recombination on the *Dro1/Ccdc80* locus by Cre recombinase in the intestinal epithelium was verified by PCR analysis (Supplementary Figure 1A). Survival was similar in *Dro1^fl/fl^;VillinCre^+^;Apc^Min/+^* mice and *Dro1^fl/fl^;Apc^Min/+^* controls (Figure 1A). Also, we found no differences in small intestinal as well as colon tumor number (Figure 1B) and tumor histology (Figure 1C). In both genotypes ~90% of colon tumors were benign adenoma and ~10% had progressed to intramucosal adenocarcinoma (Supplementary Table 1).

To further verify the initial observation of non-tumor-cell-autonomous tumor suppression by *Dro1/Ccdc80*, MC38 colorectal cancer cells reexpressing *Dro1/Ccdc80* (MC38-DRO1) and MC38-mock cells were injected subcutaneously into C57BL/6 mice. Consistent with our observations in *Apc^Min/+^* mice, growth (Figure 1D) and histology (Supplementary Figure 1B) of xenograft tumors were unaffected by *Dro1/Ccdc80* expression in tumor cells.

### Host *Dro1/Ccdc80* suppresses growth of xenograft tumors

**Figure 2:**
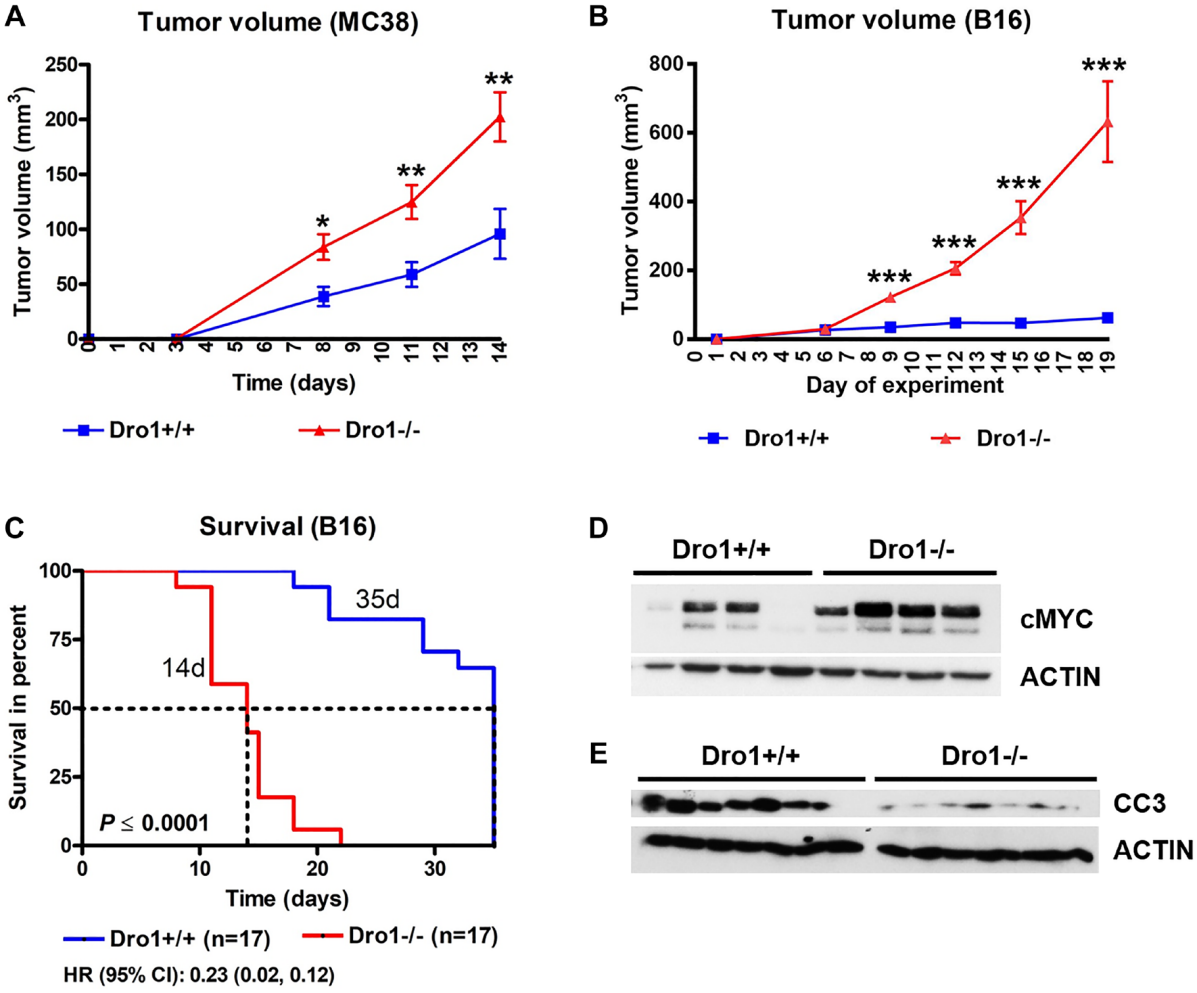
Host Dro1/Ccdc80 suppresses growth of xenograft tumors. (**A**) Tumor growth of parental MC38 colorectal cancer cells subcutaneously injected into *Dro1^-/-^* and *Dro1^+/+^* control mice (*n* = 21 tumors from 12 *Dro1^−/−^* mice and *n* = 13 tumors from 8 *Dro1^+/+^* control mice). (**B**) Tumor growth of B16 melanoma cells subcutaneously injected into *Dro1^−/−^* and *Dro1^+/+^* control mice (*n* = 36 tumors from 18 *Dro1^−/−^* mice and *n* = 31 tumors from 18 *Dro1^+/+^* control mice). (**C**) Survival of *Dro1^−/−^* and *Dro1^+/+^* control mice after subcutaneous injection of B16 melanoma cells (*n* = 17/group). (**D**, **E**) Immunoblotting for indicated proteins on whole protein lysates from B16 xenograft tumors from *Dro1^−/−^* and *Dro1^+/+^* control mice. Error bars represent standard deviations. ^*^ *p* < 0.05; ^**^ *p* < 0.01; ^***^ *p* < 0.001.

To study the effect of microenvironmental *Dro1/Ccdc80* on colon tumorigenesis, we injected parental MC38 colorectal cancer cells subcutaneously into *Dro1^−/−^* and *Dro1^+/+^* control mice. Growth of MC38 xenograft tumors was significantly increased in *Dro1^−/−^* mice compared to *Dro1^+/+^* controls (Figure 2A). However, tumor incidence was unchanged by host *Dro1/Ccdc80* inactivation (88% in *Dro1^−/−^* vs. 83% in *Dro1^+/+^* mice). Similar results were obtained using B16 melanoma cells (Figure 2B; B16 tumor incidence: 100% in both *Dro1^−/−^* and *Dro1^+/+^* mice). Moreover, when injecting B16 melanoma cells survival was dramatically reduced in the *Dro1^−/−^* group due to the fast and aggressive tumor growth (Figure 2C). Histopathological evaluation of MC38 and B16 xenograft tumors revealed no morphologic differences between tumors from *Dro1^−/−^* and *Dro1^+/+^* control mice (Supplementary Figure 2A and 2B).

Previously we have shown activation of both pERK1/2 and oncogenic c-MYC in colon tumors from *Apc^Min/+^* mice upon ubiquitous inactivation of *Dro1/Ccdc80* [1]. To investigate whether host DRO1/CCDC80 deficiency also impacts pERK1/2 and c-MYC status, immunoblot analysis of xenograft tumors was performed. No significant changes of pERK1/2 protein level were observed in B16 xenograft tumors upon microenvironmental DRO1/CCDC80 loss (Supplementary Figure 2C). However, consistent with our findings in *Apc^Min/+^* mice, c-MYC oncogene was up-regulated in xenograft tumors from *Dro1^−/−^* mice compared to *Dro1^+/+^* controls (Figure 2D). Moreover, depletion of host DRO1/CCDC80 resulted in reduced apoptosis in B16 tumors, as demonstrated by immunoblot analysis for cleaved-caspase-3 (Figure 2E).

### Stromal DRO1/CCDC80 promotes apoptosis of cancer cells

**Figure 3:**
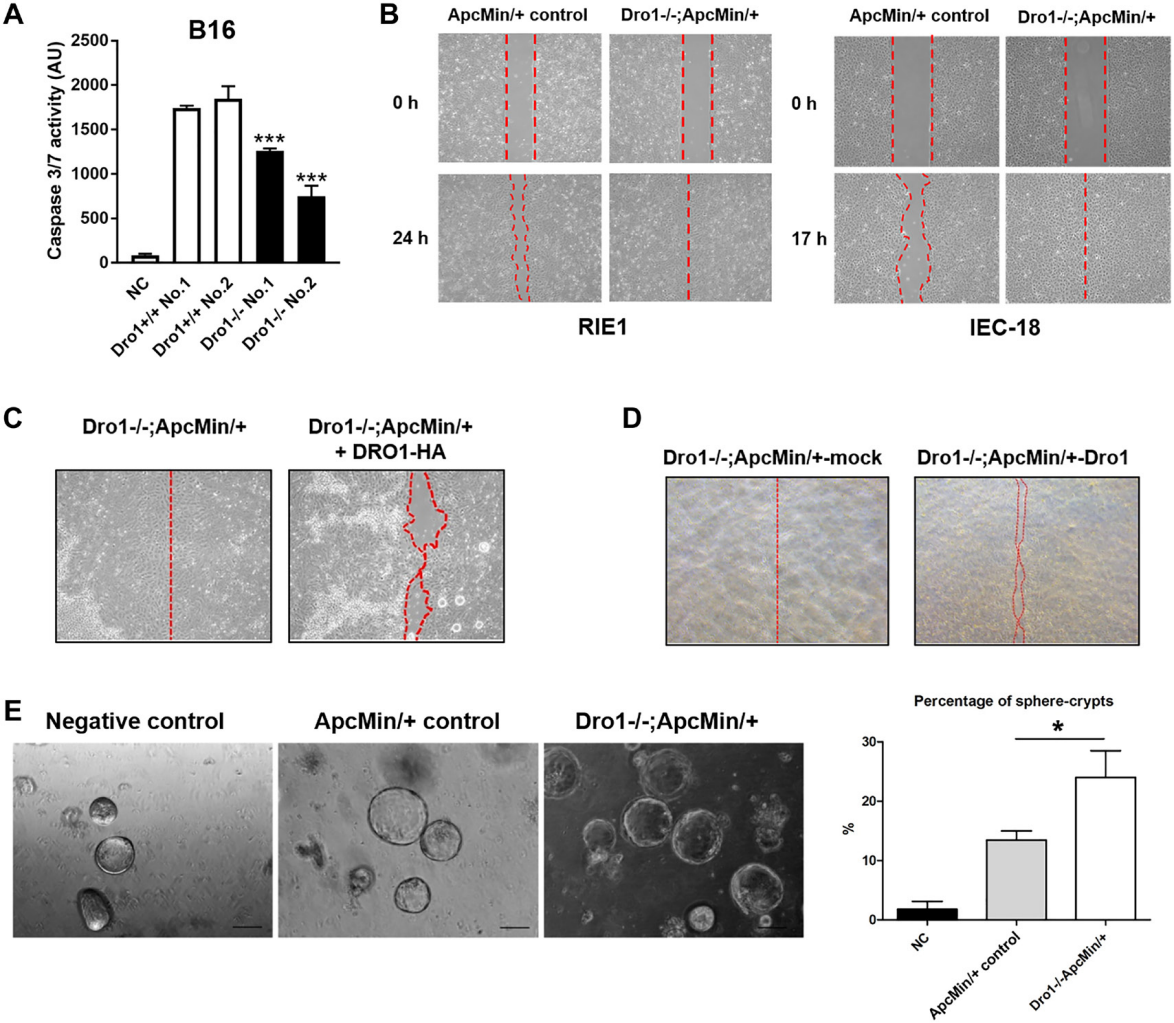
Inactivation of Dro1/Ccdc80 in stromal cells inhibits cancer cell apoptosis and promotes migration and sphere crypt formation of intestinal epithelial cells. (**A**) Caspase 3/7 activity in B16 melanoma cells 27 hours after induction of apoptosis by UVB radiation. B16 cells were treated with conditioned medium from primary stromal cells generated from B16 xenograft tumors from *Dro1^−/−^* and *Dro1^+/+^* control mice. For negative control (NC) medium was used. Measurement was performed in triplicates. (**B**) Wound scratch assays. RIE1 and IEC-18 intestinal epithelial cells were treated with conditioned medium from primary stromal cells generated from the tumor-free colon of 5-week-old *Apc^Min/+^* control and *Dro1^−/−^;Apc^Min/+^* mice. (**C**) Wound scratch assay. Migration of RIE1 cells treated with conditioned medium from primary stromal cells generated from the tumor-free colon of 5-week-old *Dro1^−/−^;Apc^Min/+^* mice. In the *Dro1^−/−^;Apc^Min/+^*+DRO1-HA group the conditioned medium was supplemented with DRO1/CCDC80-HA. (**D**) Wound scratch assay. RIE1 cells were treated with conditioned medium from *Dro1^−/−^;Apc^Min/+^*-*mock* and *Dro1^−/−^;Apc^Min/+^*-*DRO1* primary stromal cells. *Dro1^−/−^;Apc^Min/+^* primary stromal cells were generated from the tumor-free colon of 5-week-old *Dro1^−/−^;Apc^Min/+^* mice. (**E**) Sphere crypt assay. Small intestinal crypts were treated with conditioned medium from primary stromal cells generated from tumor-free colon of 5-week-old *Apc^Min/+^* control and *Dro1^−/−^;Apc^Min/+^* mice. Representative pictures of sphere crypts are shown (Scale bars, 100 μm). Percentage of sphere crypts was determined. Error bars represent standard deviations. ^*^ *p* < 0.05; ^***^ *p* < 0.001.

Since loss of host DRO1/CCDC80 strongly decreased cleavage of caspase-3 in B16 xenograft tumors, we investigated the effect of *Dro1/Ccdc80* inactivation in stromal cells on apoptosis of B16 cancer cells *in vitro*. Therefore, primary stromal cells (PSC) were generated from B16 xenograft tumors from *Dro1^−/−^* and *Dro1^+/+^* control mice. B16 melanoma cells were treated with *Dro1^−/−^* and *Dro1^+/+^* PSC conditioned medium (CM), respectively, and applied to UVB radiation for induction of apoptosis. Consistent with the *in vivo* findings, *Dro1^−/−^* PSC CM significantly reduced caspases-3/7 activity in B16 melanoma cells compared to *Dro1^+/+^* control PSC CM (Figure 3A).

### Stromal DRO1/CCDC80 inhibits migration of intestinal epithelial cells

To study the effect of stromal *Dro1/Ccdc80* on intestinal epithelial cells, PSC were generated from tumor-free colon from 5-week-old *Dro1^−/−^;Apc^Min/+^* and *Apc^Min/+^* control mice. Microscopic examination of PSC revealed no morphological differences upon DRO1/CCDC80 loss (data not shown).

*Dro1^−/−^;Apc^Min/+^* PSC CM had no effect on cellular proliferation of both RIE1 intestinal epithelial and MC38 colorectal cancer cells compared to *Apc^Min/+^* control CM (Supplementary Figure 3). Consistently, loss of DRO1/CCDC80 in PSC did not affect the growth of three-dimensional tumor spheroids generated by aggregation of MC38 colorectal cancer cells and PSC (Supplementary Figure 4A). Similar results were obtained using B16 melanoma cells (Supplementary Figure 4B).

Also, no effect of stromal *Dro1/Ccdc80* on apoptosis of intestinal epithelial cells was observed as demonstrated by unchanged caspase-3/7 activity in RIE1 cells after treatment with PSC CM and UVB radiation (Supplementary Figure 5).

*Dro1/Ccdc80* has been demonstrated to inhibit melanoma cancer cell migration [4]. To analyze the effect of stromal DRO1/CCDC80 on epithelial cell migration wound scratch assays were performed. *Dro1^−/−^;Apc^Min/+^* PSC CM increased migration of both RIE1 and IEC18 intestinal epithelial cells compared to *Apc^Min/+^* control CM (Figure 3B). To determine whether DRO1/CCDC80 protein inhibits migration of intestinal epithelial cells *Dro1^−/−^;Apc^Min/+^* PSC CM was supplemented with DRO1/CCDC80 isolated from CM of SW480 *Dro1/Ccdc80* over-expressing cells. Addition of DRO1/CCDC80 to the *Dro1^-/-^;Apc^Min/+^* PSC CM inhibited migration of RIE1 cells compared to unmodified *Dro1^−/−^;Apc^Min/+^* PSC CM (Figure 3C). Consistently, re-expression of *Dro1/Ccdc80* in *Dro1^−/−^;Apc^Min/+^* PSC resulted in reduced migration of RIE1 cells when treated with the PSC CM (Figure 3D). In contrast, *Dro1^−/−^;Apc^Min/+^* PSC CM did not affect invasion of both RIE1 and MC38 cells into a basement coated membrane compared to *Apc^Min/+^* control PSC CM (Supplementary Figure 6).

Ubiquitous inactivation of *Dro1/Ccdc80* leads to increased phosphorylation of ERK1/2 and oncogenic c-MYC activation in colon tumors from *Apc^Min/+^* mice [1]. Moreover, in the present study, we found loss of host DRO1/CCDC80 to increase c-MYC protein levels in xenograft tumors (Figure 2D). To investigate whether stromal inactivation of *Dro1/Ccdc80* influences MAP kinase signaling and c-MYC levels in intestinal epithelial cells *in vitro*, RIE1 cells were treated with PSC CM and immunoblot analysis was performed. No significant differences in pERK1/2, ERK1/2, pP70S6K, P70S6K, and c-MYC protein levels were observed in RIE1 cells when treated with the CM from *Dro1^−/−^;Apc^Min/+^* and *Apc^Min/+^* control PSC, respectively (Supplementary Figure 7).

### Stromal DRO1/CCDC80 affects formation of intestinal organoids

Epithelial loss of DRO1/CCDC80 had no effect on colon tumor development (Figure 1A–1D). To further investigate the role of epithelial *Dro1/Ccdc80* in colon tumorigenesis, specifically in stemness, we established epithelial organoid cultures from colon tumors that were harvested from *Dro1^−/−^;Apc^Min/+^* and *Apc^Min/+^* control mice. *In vitro*-generated *Dro1^−/−^;Apc^Min/+^* and *Apc^Min/+^* control colon tumor organoids were growing as cystic structures (Supplementary Figure 8A). Diameter of *Dro1^−/−^;Apc^Min/+^* colon tumor organoids was significantly decreased when compared to *Apc^Min/+^* control organoids (207.2 ± 13.85 μm vs. 265.4 ± 17.88 μm; *p* = 0.0119; Supplementary Figure 8B).

Next we tested the effect of microenvironmental *Dro1/Ccdc80* on the formation of intestinal organoids. Therefore, wildtype small intestinal crypts were treated with *Dro1^-/-^;Apc^Min/+^* and *Apc^Min/+^* PSC CM, respectively. The percentage of developing spheroides was significantly increased in the *Dro1^−/−^;Apc^Min/+^* PSC group compared to the *Apc^Min/+^* control group (Figure 3E).

### Down-regulation of *Dro1/Ccdc80* in the stromal tumor compartment

**Figure 4:**
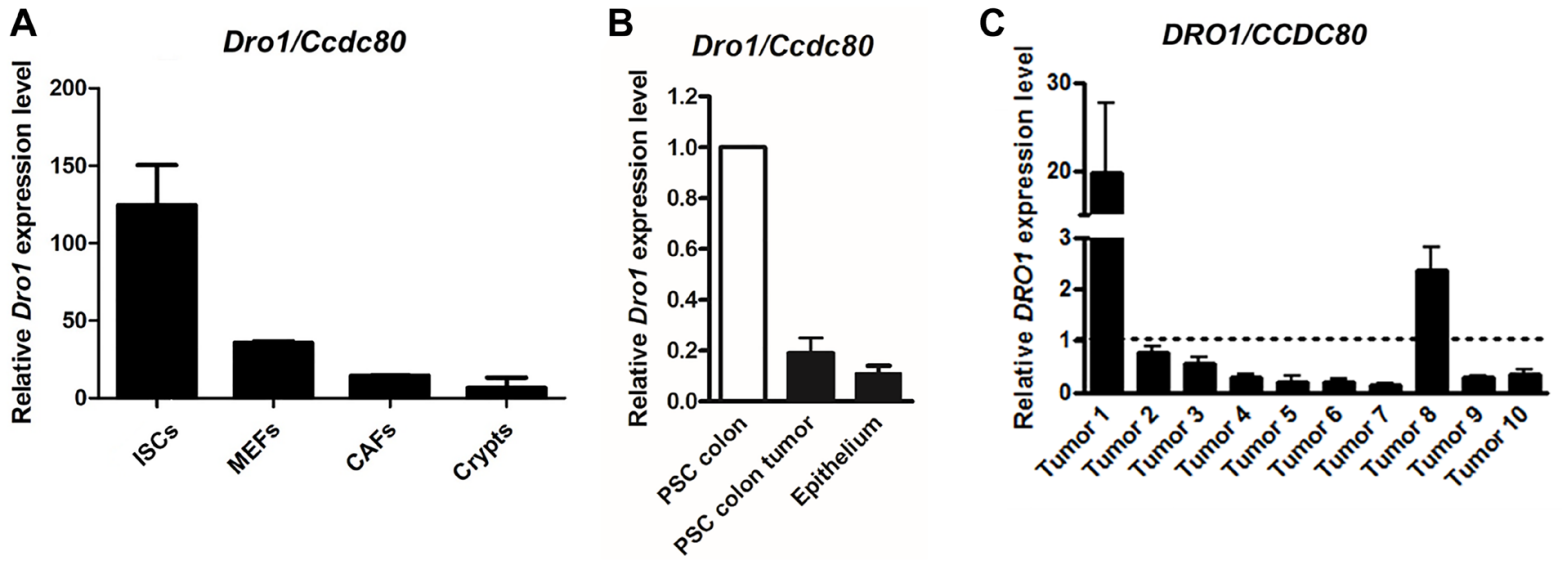
DRO1/CCDC80 is down-regulated in the stromal tumor compartment. (**A**) Relative *Dro1/Ccdc80* mRNA expression in C57BL/6 mouse small intestinal stromal cells (ISCs), mouse embryonic fibroblasts (MEFs; day 18 p.c.), mouse gastric cancer associated fibroblasts (CAFs), and mouse small intestinal crypts (Crypts). (**B**) Relative *Dro1/Ccdc80* mRNA expression in primary stromal cells generated from tumor-free colon from 5-week-old *Apc^Min/+^* mice and from colon tumors from moribund *Apc^Min/+^* mice and in scratched colon epithelium from 5-week-old *Apc^Min/+^* mice. *DRO1/CCDC80* expression in PSC from colon tumor and from epithelium is represented relative to expression in normal PSC (set to 1). (**C**) Relative *DRO1/CCDC80* mRNA expression in microdissected human primary tumor stroma from colorectal carcinoma specimens compared to microdissected normal colorectal connective tissue. Matched pairs of tumor stroma and normal adjacent colorectal stroma from 10 patients were analyzed. *DRO1/CCDC80* expression in colorectal carcinoma stroma is represented relative to expression in normal stroma (set to 1, see dotted line). Error bars represent standard deviations.

*Dro1/Ccdc80* has been demonstrated to be expressed in various tissues including small intestine and colon [1, 2, 16]. Since former expression analyses utilized RNA isolated from whole small intestinal and colonic tissue samples, we now investigated expression of *Dro1/Ccdc80* separately in the epithelial and stromal compartment. In small intestinal crypts from C57BL/6 mice and in scratched colon epithelium from *Apc^Min/+^* mice *Dro1/Ccdc80* expression was very low (Figure 4A and 4B). On the contrary, when the stromal compartment was studied, *Dro1/Ccdc80* mRNA level was abundant in C57BL/6 small intestinal stromal cells, mouse embryonic fibroblasts, and murine gastric cancer associated fibroblasts (Figure 4A). Remarkably, in cancer associated fibroblasts *Dro1/Ccdc80* expression was highly down-regulated compared to normal intestinal stromal cells (Figure 4A). Consistently, *Dro1/Ccdc80* expression was strongly reduced in PSC isolated from colon tumors from moribund *Apc^Min/+^* mice compared to PSC generated from tumor-free colon from 5-week-old *Apc^Min/+^* mice (Figure 4B).

Previously, we have demonstrated *DRO1/CCDC80* to be down-regulated in the majority of primary human colorectal carcinoma specimens [1, 2]. Since *Dro1/Ccdc80* mRNA level was found to be reduced in murine cancer associated fibroblasts, we investigated expression of *DRO1/CCDC80* in micro-dissected stroma from human primary colorectal cancer compared to expression in adjacent normal intestinal stroma. Microenvironmental down-regulation of *DRO1/CCDC80* was found in 80% of human colorectal cancer specimens (Figure 4C).

## DISCUSSION

In the present study we demonstrate that tumor suppression by *Dro1/Ccdc80* is not tumor-cell-autonomous but is mediated by the tumor microenvironment. Colon tumor development in *Apc^Min/+^* mice as well as formation of xenograft tumors was unaffected by *Dro1/Ccdc80* expression in cancer cells, suggesting epithelial derived DRO1/CCDC80 to be dispensable for intestinal tissue homeostasis. In contrast, mutational inactivation of host *Dro1/Ccdc80* highly promotes growth of xenograft tumors, suggesting a strong tumor suppressive role for microenvironmental *Dro1/Ccdc80*.

In B16 xenograft tumors inactivation of host *Dro1/Ccdc80* resulted in dramatically reduced cleavage of caspase-3. Consistently, conditioned medium from *Dro1/Ccdc80* deficient PSC from B16 xenograft tumors significantly inhibited caspase-3/7 activity in apoptosis induced B16 cancer cells. Thus, our data point to microenvironmental *Dro1/Ccdc80* as a promoter of cellular apoptosis. Evasion from apoptosis is a prominent hallmark of cancer cells and fundamental for cancer development [17]. Previously, we have found *Dro1/Ccdc80* to sensitize cancer cells to various apoptotic stimuli [2, 18]. In particular, *DRO1/CCDC80* sensitizes cells to receptor-mediated apoptosis [2, 18]. Moreover, *DRO1/CCDC80* significantly impairs cell survival under anchorage independent growth conditions by induction of detachment induced apoptosis (anoikis) [2]. In mammary carcinoma cells carrying an oncogenic activation of *AIB1*, inhibition of apoptosis has been shown to be at least partly mediated by repression of *DRO1/CCDC80* expression [19]. Also, mouse embryonic fibroblasts generated from *Dro1/Ccdc80* null mice are less sensitive to apoptotic stimuli with respect to mouse embryonic fibroblasts generated from wildtype mice [3].

In contrast to our findings in B16 melanoma cells, CM from *Apc^Min/+^* *Dro1/Ccdc80* *knockout* PSC did not affect activity of caspase-3/7 in apoptosis induced intestinal epithelial cells, suggesting no modulatory role for DRO1/CCDC80 in apoptosis in the intestinal epithelium. Consistently, we have found no effect of ubiquitous inactivation of *Dro1/Ccdc80* on the rate of cellular apoptosis in the intestinal epithelium and in colon tumors from *Apc^Min/+^* mice [1]. Moreover, we could observe no influence of ubiquitous DRO1/CCDC80 loss on the apoptotic rate in carcinogen-induced colonic neoplastic lesions [6]. Thus, *Dro1/Ccdc80*’s function in cellular apoptosis might be tissue dependent. Future studies are needed to further elucidate the role of *Dro1/Ccdc80* in the apoptotic process during cancer development.

Activation of the c-MYC oncogene is a common molecular hallmark of many cancers contributing to both tumor initiation and progression [20]. We found depletion of host DRO1/CCDC80 to result in c-MYC oncogenic activation in xenograft tumors, suggesting microenvironmental loss of DRO1/CCDC80 to drive carcinogenesis by c-MYC. Consistently, we demonstrated ubiquitous inactivation of *Dro1/Ccdc80* to implicate increased c-MYC protein levels in the colonic epithelium and in colon tumors from *Apc^Min/+^* mice [1]. However, treatment of RIE1 cells with CM from *Dro1/Ccdc80* *knockout* PSC had no impact on c-MYC protein level. In contrast to tumor tissue, RIE1 cells are non-tumorigenic intestinal epithelial cells [21]. Thus, the effect of DRO1/CCDC80 loss in stromal cells on c-MYC activation in epithelial cells might depend on second pro-tumorigenic mutational hits in epithelial cells. Similarly, despite *Dro1/Ccdc80*’s strong tumor suppressive function in *Apc^Min/+^* mice as well as chemically-induced colon carcinogenesis [1, 6], ubiquitous loss of DRO1/CCDC80 alone was insufficient to induce spontaneous colonic tumor development in C57BL/6 mice [1].

Our results show inactivation of *Dro1/Ccdc80* in stromal cells to stimulate migration of intestinal epithelial cells, indicating an important role for microenvironmental *Dro1/Ccdc80* as regulator of epithelial cell motility. Previously, *Dro1/Ccdc80* has also been shown to inhibit melanoma cancer cell migration [4]. Modulation of migratory properties of epithelial cells is implicated in many physiological processes, such as embryogenesis and wound healing, but also in cancer development [22]. During cancer progression, the tumor stroma has been shown to play a pivotal role in the control of migration, invasion, and finally metastasis of cancer cells by secretion of a plethora of molecules, including chemokines, cytokines, and growth factors [23]. Thus, loss of *Dro1/Ccdc80* function in the tumor stroma might promote cancer progression by creating a permissive environment for cancer cell migration. Interestingly, we could observe no effect of stromal *Dro1/Ccdc80* inactivation on invasive properties of intestinal epithelial cells, suggesting *Dro1/Ccdc80* to control cell migration but not cell invasiveness.

Our data also indicate DRO1/CCDC80 to be an extracellular matrix (ECM) protein since the pro-migratory effect of *Dro1/Ccdc80* loss in stromal cells on intestinal epithelial cells could be partly reversed by supplementation with DRO1/CCDC80 protein. A crucial role in cancer progression has been attributed to the ECM, which regulates cell behavior, tissue homeostasis, cell adhesion, and motility [22, 23]. Previously, DRO1/CCDC80 has been shown to be a secreted protein in various cell types [16, 24–26] and it has been identified as an ECM protein involved in matrix assembly and ECM molecule binding [25]. Moreover, DRO1/CCDC80 has been shown to be implicated in cell adhesiveness [25, 27]. Adhesion of chick lens cells and various tumor cell lines has been demonstrated to be mediated by DRO1/CCDC80 through heparin sulfate proteoglycan [27]. During chicken lens development extracellular DRO1/CCDC80 promotes lens cell adhesion and migration by activation of FGF signaling [27]. In contrast, we could observe no effect of *Dro1/Ccdc80* loss in stromal cells on FGF signaling in intestinal epithelial cells as demonstrated by unchanged pERK1/2 levels. Our data support the hypothesis of DRO1/CCDC80 being an extracellular matrix protein that modifies epithelial cell migration, however, the molecular mechanisms by which DRO1/CCDC80 inhibits intestinal epithelial cell migration have to be elucidated.

*Dro1/Ccdc80* inactivation did not improve growth of epithelial organoids derived from *Apc^Min/+^* colon tumors, providing further evidence that the tumor suppressive function of DRO1/CCDC80 is not tumor-cell-autonomous but dictated by extrinsic cues provided by the local tissue microenvironment. In contrast, stromal *Dro1/Ccdc80* inactivation increased sphere crypt forming ability of small intestinal organoids. Since intestinal organoids, known as mini-gut cultures, are maintained *in vitro* due to the presence of stem cells [28], we speculate that stromal cell-derived DRO1/CCDC80 may negatively regulate Lgr5+ stem cells, or Cnx43+ progenitors [29]. Since loss of stromal DRO1/CCDC80 facilitates migration of intestinal epithelial cells, stromal DRO1/CCDC80 might probably regulate transition from a stationary cancer stem cell into a migrating cancer stem cell during the multistep progression of colorectal cancer. Future studies are required to further investigate *Dro1/Ccdc80*’s role in cancer stemness.

We found *Dro1/Ccdc80* to be significantly down-regulated in *Apc^Min/+^* colon tumor primary stromal cells and in microdissected stroma from human colorectal cancer compared to normal, non-tumor stroma. Moreover, *Dro1/Ccdc80* expression was reduced in gastric cancer associated fibroblasts with respect to normal intestinal stromal cells. Alterations in the tumor microenvironment are supposed to contribute to cancer initiation, progression, invasion, and metastasis [8–12]. Dramatic changes in gene expression patterns have been demonstrated for all cell types of the tumor microenvironment [30]. Several studies have shown genetic alterations in tumor-associated stromal cells, including somatic mutations in key tumor suppressor genes [31–39]. Frequent mutations in *TP53* have been found in the stromal compartment of breast cancer and colorectal carcinoma [34–36]. *PTEN* has been demonstrated to be mutated in stromal cells of breast cancer [34] and somatic alterations in the *GT198* tumor suppressor have been found in ovarian tumor stromal cells of various types of human ovarian cancer [40]. For breast and prostate cancer changes in microenvironmental gene expression patterns have also been shown to be at least partly due to distinct epigenetic modifications [39, 41, 42]. The data from the present study suggest microenvironmental loss of *Dro1/Ccdc80*’s tumor suppressive function to be an important event during carcinogenesis.

In summary, we identify *Dro1/Ccdc80* as tumor suppressor in the tumor microenvironment. DRO1/CCDC80 in the stromal compartment strongly inhibited tumor growth, facilitated apoptosis in cancer cells, and reduced epithelial cell migration. Moreover, stromal DRO1/CCDC80 restrained the formation of epithelial organoids, indicating a possible role for *Dro1/Ccdc80* in stemness. Our study provides new insights into the complex interaction between epithelial cells and their microenvironment and contributes to the understanding of cancer development. Future studies are needed to further elucidate the molecular mechanisms underlying *Dro1/Ccdc80’s* microenvironmental tumor suppressive function and to better characterize its role in human cancer.

## MATERIALS AND METHODS

### Animals

*Dro1^−/−^* mice were generated as described previously [1]. Mice on the *Dro1*^fl/fl^ background (in the following referred to as *Dro1*^+/+^ mice) were used as controls.

*Apc*^Min/+^ mice were purchased from the Jackson Laboratory and maintained on a C57BL/6J background. Mice were inspected on a daily basis and sacrificed when moribund.

Animals were housed under specific pathogen free conditions in a closed barrier system. All mice had access to water and to the same standard rodent diet (V1534, Ssniff, Soest, Germany) *ad libitum*. Experiments were carried out in accordance with the German Animal Welfare Act and with permission of the Government of Upper Bavaria (AZ55.2-1-54-2532-25-11 and AZ55.2-1-54-2532-48-2015).

### PCR

Mice were genotyped by PCR analysis of genomic DNA from tail tip samples as described previously [1]. For purification of total RNA from microdissected FFPE tissue sections from human colorectal carcinoma specimens and from normal colon stroma, the RNeasy FFPE kit (Qiagen, Germany) was used. For quantitative RT-PCR RNA cleanup was performed using the RNeasy Mini kit (Qiagen, Germany). Genomic DNA was removed from the RNA preparation using DNase I, Amplification Grade (Invitrogen, USA). First-strand cDNA was generated using the SuperScript First Strand cDNA Synthesis System (Invitrogen, USA). Primer sequences for quantitative RT-PCR are displayed in Supplementary Table 2.

### Tumor scoring and histology

Mice were sacrificed by cervical dislocation. The small intestine was rinsed with PBS, cut into 3 equal segments and each intestinal section was placed on a piece of filter paper, opened longitudinally, laid open and fixed in 4% buffered formaldehyde solution. Tumor number and their maximum diameter were determined under a dissecting microscope at 10× magnification. The colon and rectum were scored as “colon”. A quantity of small intestinal lesions and all colonic tumors sized ≥ 2 mm in diameter were resected including adjacent normal tissue, dehydrated and embedded in paraffin, 4 μm tissue sections cut in parallel with the mucosal surface and stained with hematoxylin and eosin (H&E). Histopathologic analysis of neoplastic lesions was performed in a blinded manner using standard criteria according to the classification of human adenomas of the colon and the assessment of the degree of dysplasia. The diagnosis intramucosal adenocarcinoma was made for lesions with high grade dysplasia/intraepithelial neoplasia (IEN) in combination with focal invasion of the lamina propria mucosae, cytologic features such as cribriform architecture with intraluminal accumulation of tumor and inflammatory cell debris (dirty necrosis) and desmoplastic stomal reaction. Adenocarcinomas invading through the lamina muscularis mucosae into the tela submucosa were classified as invasive adenocarcinoma [43]. Invasive adenocarcinoma and intramucosal adenocarcinoma were summarized under the diagnosis adenocarcinoma.

For xenograft experiments mice were sacrificed by cervical dislocation, the tumors excised, fixed in 4% buffered formaldehyde solution, and dehydrated and embedded in paraffin. 4 μm tissue sections were cut and stained with H&E. Histopathologic analysis of neoplastic lesions was performed in a blinded manner.

### Xenograft experiments

Mice were injected subcutaneously with 10^6^ tumor cells (MC38, B16) in 100 μl PBS. Each mouse received two injections (right and left flank). Mice were inspected every day and size of xenograft tumors was measured twice a week. Mice were sacrificed when one of the following endpoints was reached: tumor diameter ≥ 1.5 cm; penetration of tumor through the skin; moribund state. Tumors were dissected and the tumor weight determined.

### Transfection of MC38 cells

MC38 cells were transfected with plasmids pCDNA3-DRO1-HA [2] and pCDNA3-mock for control. Transfection was performed as described before [44].

### Immunoblotting

Protein lysates from mouse tissue samples were generated using M-PER mammalian extraction reagent (Thermo Scientific, USA) and separated by electrophoresis in discontinuous SDS-polyacrylamide gels. The following antibodies were used for immuno detection: pERK1/2 (Cell Signaling, USA), ERK1/2 (Cell Signaling, USA), c-MYC (Cell Signaling, USA), Cleaved-Caspase-3 (Cell Signaling, USA), pP70S6K (Cell Signaling, USA), P70S6K (Cell Signaling, USA), and anti-ACTIN (MP Biomedicals, USA).

### Isolation of primary stromal cells

For generation of primary stromal cells from tumor-free whole colon samples, the colon from 5-week-old mice was cleaned from fecal debris and cut into 2–3 mm^3^ pieces and washed with Hank’s Balanced Salt Solution (HBSS, Life Technologies, USA). For generation of primary stromal cells from tumors, the tumors were excised, and cut into 2–3 mm^3^ pieces and washed with Hank’s Balanced Salt Solution (HBSS, Life Technologies, USA). The tissue pieces (colon/colon tumors) were incubated with 1 mM dithiothreitol (DTT, Sigma). After that, tissue pieces were incubated in 1 mM EDTA, followed by washing with HBSS. Then, incubation in 1 mM EDTA was repeated, and tissue pieces were washed with HBSS, followed by incubation in 1 mg/ml collagenase type I solution (Sigma). After washing in HBSS, tissue pieces were spun down and cultured in 10 cm Petri dishes in primary stromal cell medium (PSC medium) composed of: RPMI1640 (Life Technologies), 10% FBS, 1% penicillin/streptomycin (10,000 Units/ml penicillin; 10,000 μg/ml streptomycin; Life Technologies) and 100 μg/ml Normocin (Invivogen). Primary stromal cells migrated out of the tissue fragments and adhered to the plates.

In primary stromal cell cultures absence of epithelial cells was verified by immunofluorescence analysis for E-Cadherin. Primary stromal cells were also stained for α-SMA. For immunofluorescence primary stromal cells were grown on CELLview slides (Greiner Bio-One, Germany). Cells were fixed with 4% paraformaldehyde and permeabilized in 0.1% Triton X-100 (Sigma). First antibodies were diluted 1: 100 for mouse-anti-E-Cadherin (BD) and 1: 200 for rabbit-anti-α-SMA (ACTA2, proteintech). Second antibodies were donkey anti-mouse IgG (H+L) highly cross-adsorbed antibody conjugated with Alexa Fluor 488 (Invitrogen, USA) for E-Cadherin and donkey anti-rabbit IgG antibody conjugated with Alexa Fluor 647 (Jackson ImmunoResearch) for α-SMA diluted 1: 1000. Slides were mounted using VECTASHIELD Mounting Medium with DAPI (Vector Laboratories, USA). Fluorescence microscopy was performed on a Leica microscope.

### Apoptosis assay (caspase-3/7 activity)

10.000 cells (RIE1, B16) were seeded per well of a 96-well-plate and incubated with conditioned medium from primary stromal cells for 24 h and subsequently applied to 450 mJ/cm^2^ UVB radiation [medisun HF-144 (Schulze & Böhm, Germany)] for induction of apoptosis. For negative control medium [DMEM (Sigma) containing 10% FCS and 1% penicillin/streptomycine] was used. After UVB radiation cells were again incubated with primary stromal cell conditioned medium or medium. 27 h after UVB radiation activity of caspase-3/7 was measured using the Apo-ONE Homogeneous caspase-3/7 assay (Promega, USA) according to the manufacturer’s manual. Experiments were performed in triplicates.

### Proliferation assay

2.000 cells (RIE1, MC38) were seeded per well of a 96-well-plate and incubated with conditioned medium from primary stromal cells for the duration of the assay. Proliferation assays were performed using the Cell Proliferation ELISA, BrdU (colorimetric) (Sigma) according to the manufacturer’s manual. Experiments were performed in triplicates.

### Generation of three-dimensional tumor spheroids

Tumor spheroids were generated by magnetic levitation [45] using the NanoShuttle^™^-PL system (Nano3D Biosciences, Inc., USA) according to the manufacturer’s manual. For labeling cells were incubated with NanoShuttle^™^-PL solution (1 μl/10.000 cells) in DMEM medium containing 0.5% FCS and 1% penicillin/streptomycin for 12 h. 125.000 labeled cancer cells (MC38, B16) and 125.000 labeled primary stromal cells per well were seeded in a 24-well cell-repellent surface plate (Greiner Bio-One, Germany). A levitating magnetic device (Nano3D Biosciences, Inc., USA) was put on top of the plate overnight. The next day, the levitating magnetic device was exchanged for a concentrating magnetic device (Nano3D Biosciences, Inc., USA) placed on the bottom of the plate and incubated overnight. The next day, the concentrating magnetic device was exchanged for the levitating magnetic device. Medium was exchanged twice a week using the concentrating magnetic device. Growth of spheroids was monitored every day under a light microscope. Pictures were taken with a Leica microscope (Type 11090137002). Size of spheroids was calculated from two-dimensional pictures using the Leica Application Suite V4.5 software. Experiments were performed in duplicates.

### Invasion assay

Invasion assays were performed by co-cultivation in a Boyden chamber using CultreCoat Medium BME Cell Invasion Assay (R&D Systems, USA) according to the manufacturer’s manual. 25.000 epithelial cells (RIE1, MC38) per well were seeded in the upper, basement coated chamber. 15.000 primary stromal cells were seeded in the lower chamber. Experiments were performed in 8 replicates.

### Wound scratch assay

Migration assays were performed in cell inserts having two separate wells divided by an insert (ibidi). After removal of the insert a defined ~500 μm cell free gap is left behind as migration area. For cultivation cell inserts were placed in 35 mm μ-dishes (ibidi). On day one 40.000 epithelial cells (RIE1, IEC-18) were seeded in DEMEM (Sigma) containing 10% FCS and 1% penicillin/streptomycine in each well of the two-well insert. On day two cultivation medium was changed to DEMEM (Sigma) containing 0.5% FCS and 1% penicillin/streptomycin. On day three cultivation medium was exchanged for conditioned medium of primary stromal cells and the insert was removed for start of cell migration. Pictures were taken at the time of removal of the insert (defined as 0 h of migration assay) and 17–24 h after removal of the insert with a Leica microscope (Type 11090137002). Experiments were performed in duplicates.

### Purification of DRO1/CCDC80

DRO1/CCDC80 was purified from conditioned medium of SW480-*Dro1-HA* overexpressing cells [2]. For control experiments conditioned medium from SW480-mock cells was used [2]. SW480-*Dro1-HA* and SW480-mock cells were grown confluent in 10 cm cell culture dishes in DMEM (Sigma) culture medium containing 10% FCS and 1% penicillin/streptomycine. DRO1/CCDC80-HA expression in SW480 cells was induced by addition of Doxycycline (10 μg/ml) to the medium for 18 h. Anti-HA antibody (1: 16; Abcam) was preincubated with 50 μl Sepharose beads (Invitrogen, USA) in 250 μl PBS for 1 h and subsequently mixed with the conditioned medium. After washing with PBS a pH-shift was induced by addition of 0.1 M glycine (pH 3.0). Neutralisation was induced by treatment with 1M Tris (pH 9.0). pH-shift and neutralization was repeated to increase DRO1/CCDC80 yield. For migration experiments DRO1/CCDC80 isolated from 10 ml SW480-DRO1-HA conditioned medium was divided equally between two migration culture plates.

### Colon tumor organoids

Colon tumors were harvested, washed with ice-cold Hank’s Balanced Salt Solution (Life Technologies) and cut into small pieces. Tissue fragments were washed with the washing solution: 10% FBS (Life Technologies) in PBS (Life Technologies); followed by incubation with 2 mM EDTA. Tissue fragments were firstly washed with the washing solution and then with the crypt basal medium (CBM; Supplementary Table 3). After that, tissue fragments were incubated with collagenase type I (1 mg/ml), washed with the washing solution, pelleted, mixed with Matrigel (BD Biosciences) and seeded in a 24-well plate. Just after the isolation, tumor crypts were cultured in a medium composed of 12.5% Wnt conditioned medium and 87.5% crypt complete medium (CCM; Supplementary Table 4), afterwards CCM was used for the culture. Wnt conditioned medium was collected from the supernatant of cultured L-Wnt3a cell line.

### Sphere crypt assay

Small intestinal crypts were isolated as previously described [28, 46]. Briefly, the small intestine was harvested from a wildtype mouse and cut into small pieces. After several washing steps, tissue fragments were incubated with 2 mM EDTA and then passed through cell strainer 70 μm. The flow-through fraction, which contained crypts, was pelleted and then Matrigel (BD Biosciences) was added. Crypts resuspended in Matrigel were plated in a 24-well plate and cultured in CCM medium (Supplementary Table 4). For the sphere crypt assay, on day 0 crypts were seeded in a new 24-well plate and incubated with conditioned medium derived from colonic primary stromal cells. On day 1, sphere crypts were quantified. Sphere crypts were defined as non-budding, round crypts composed of a thin epithelial layer. For each experimental group at least 1000 organoids were quantified.

### Cell line origin and authentication

Cell lines were obtained from Thermo Fischer Scientific (MC38, 1/2014), ATCC (IEC-18, 3/2014) and DSMZ (B16, 1/2014). Cell lines were tested and authenticated by generation of DNA profiles of eight highly polymorphic locations of Short Tandem Repeats (STRs) using a human nonaplex PCR system. In addition, the samples were tested for the presence of mitochondrial DNA sequences from rat, Syrien and Chinese Hamster. Finally, the samples were subjected to DNA Barcoding in order to identify the animal species. For mouse strain specificity murine STR technology was applied. Cell line authentication was performed by Leibniz-Institut DSMZ GmbH, Germany.

### Statistical analysis

To display the time to tumor mortality Kaplan-Meier survival curves were used and logrank statistics was employed to test for differences between genotype groups. To analyze significance of differences, two-tailed Student’s *t*-test or two-tailed Mann Whitney *U* test were performed (GraphPad Prism). Multiple comparisons were performed using 1-way ANOVA (GraphPad Prism). *P* values < 0.05 were considered to be statistically significant.

## SUPPLEMENTARY MATERIALS


