# Supplementary material for: Loss of DRO1/CCDC80 in the tumor microenvironment promotes carcinogenesis
Source: Oncotarget. 2022 Apr 11;13:615–27. doi: 10.18632/oncotarget.28084 (PMC9004603; doi:10.18632/oncotarget.28084)
Supplement: Supplementary file 1 [file oncotarget-13-28084-s001.pdf]

Loss of DRO1/CCDC80 in the tumor microenvironment promotes carcinogenesis

SUPPLEMENTARY MATERIALS

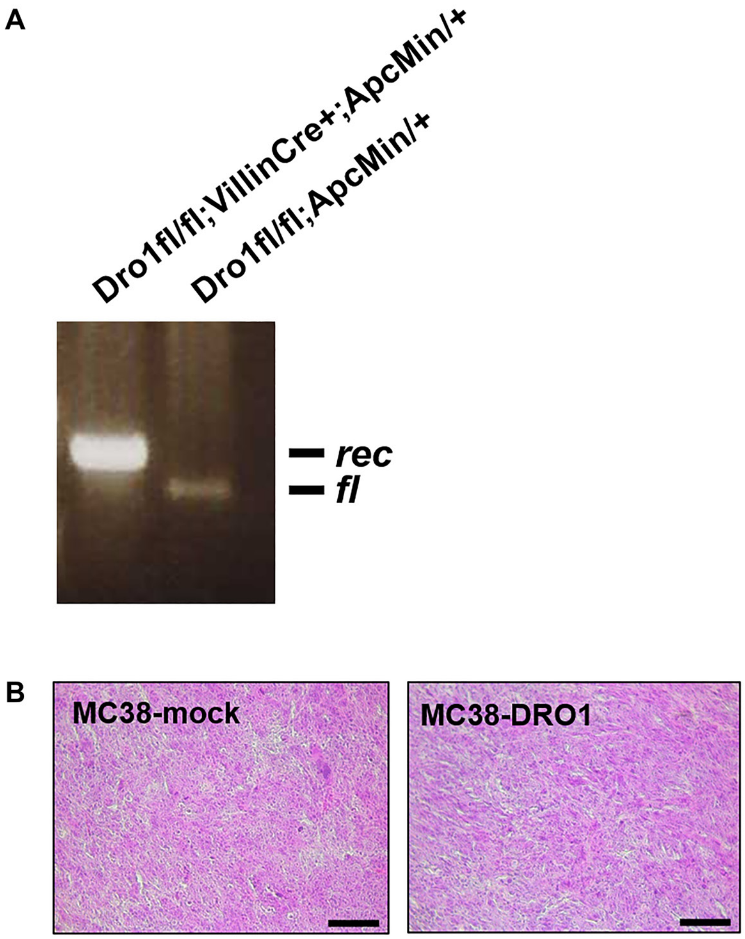

Supplementary Figure 1: (**A**) PCR analysis for the *Dro1/Ccdc80* locus. DNA was isolated from scratched intestinal epithelium from a *Dro1*$^{-/-}$;*VillinCre*$^{+}$;*Apc*$^{Min/+}$ and a *Dro1*$^{fl/fl}$;*Apc*$^{Min/+}$ control mouse. (**B**) Representative pictures of MC38-DRO1 and MC38-mock xenograft tumors from C57BL/6 mice. H&E-staining. Scale bars, 100 µm.

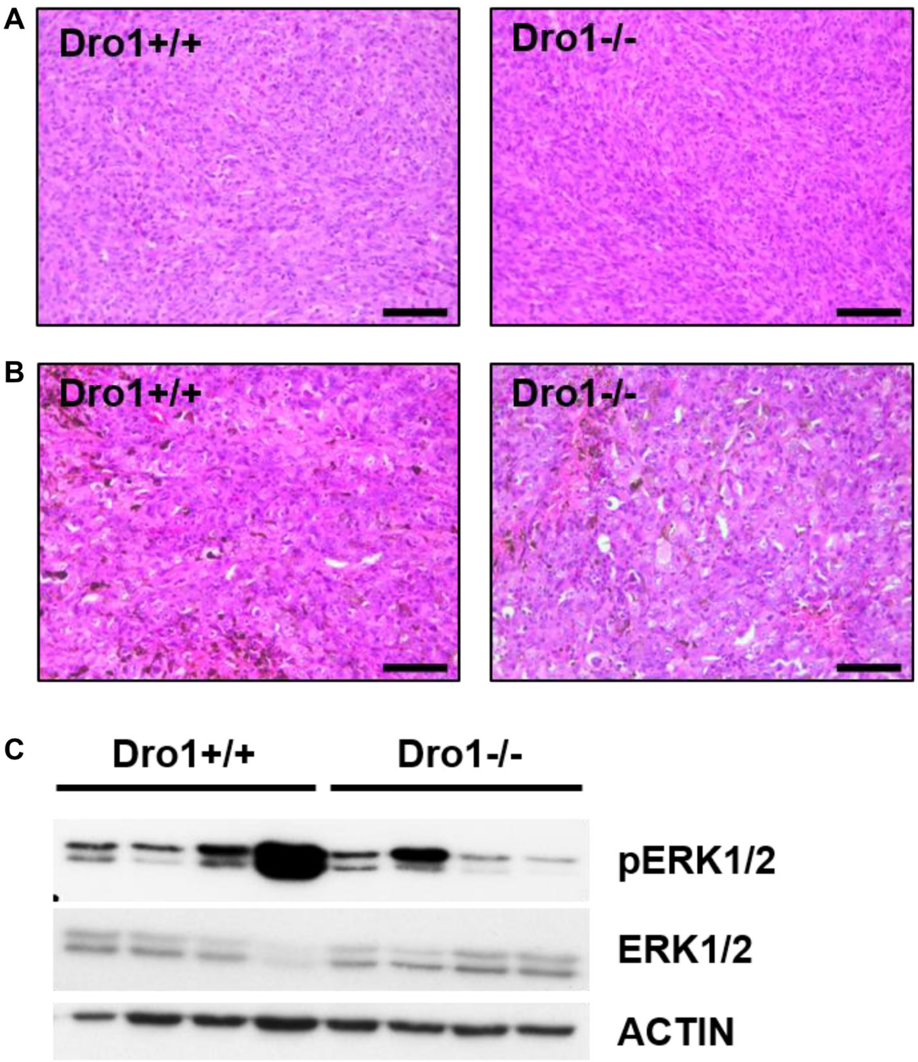

Supplementary Figure 2: (**A**) Representative pictures of MC38 xenograft tumors from a *Dro1*$^{-/-}$ and a *Dro1*$^{+/+}$ control mouse. H&E-staining. Scale bars, 100 µm. (**B**) Representative pictures of B16 xenograft tumors from a *Dro1*$^{-/-}$ and a *Dro1*$^{+/+}$ control mouse. H&E-staining. Scale bars, 100 µm. (**C**) Immunoblotting for indicated proteins on whole protein lysates from B16 xenograft tumors from *Dro1*$^{-/-}$ and *Dro1*$^{+/+}$ control mice.

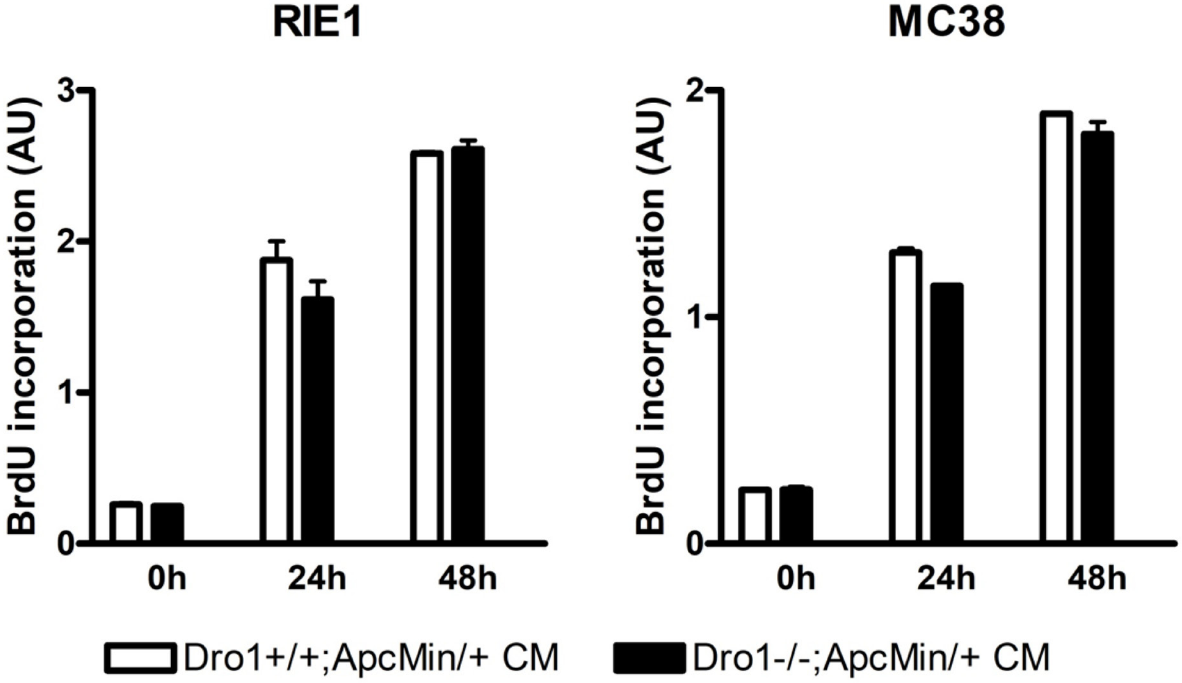

Supplementary Figure 3: Rate of proliferation of RIE1 and MC38 cells treated with conditioned medium (CM) from primary stromal cells generated from tumor-free colon from 5-week-old *Dro1*$^{-/-}$; *Apc*$^{Min/+}$ and *Apc*$^{Min/+}$ control mice (*n* = 3 biological replicates/genotype). Error bars represent standard error of mean.

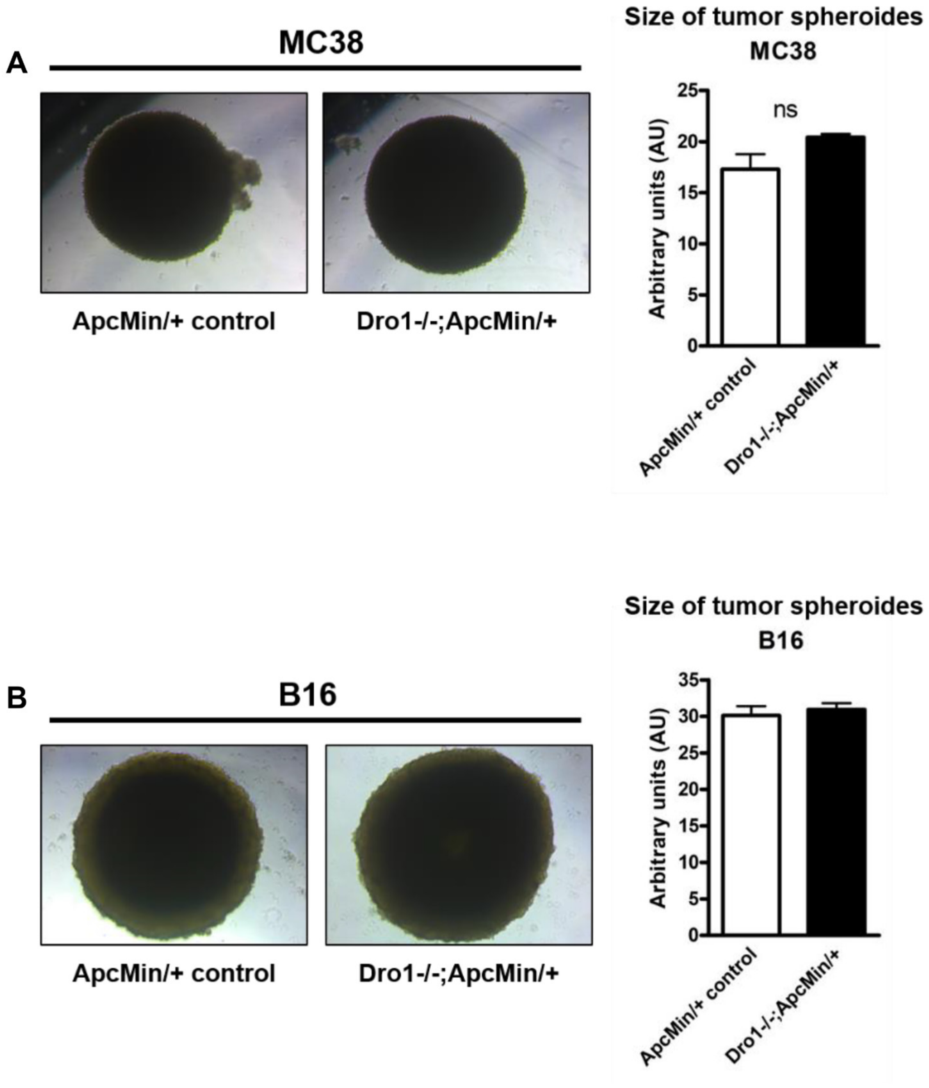

Supplementary Figure 4: Generation of three-dimensional tumor spheroids. (**A**) Representative pictures of tumor spheroids generated by aggregation of MC38 colorectal cancer cells and primary stromal cells isolated from the tumor-free colon of 5-week-old *Dro1*$^{-/-}$; *Apc*$^{Min/+}$ and *Apc*$^{Min/+}$ control mice. Size of tumor spheroids was calculated from two-dimensional pictures (n = 3 biological replicates/genotype). (**B**) Representative pictures of tumor spheroids generated by aggregation of B16 melanoma cells and primary stromal cells isolated from the tumor-free colon of 5-week-old *Dro1*$^{-/-}$;*Apc*$^{Min/+}$ and *Apc*$^{Min/+}$ control mice. Size of tumor spheroids was calculated from two-dimensional pictures (n = 3 biological replicates/genotype). AU, arbitrary units. Error bars represent standard error of mean.

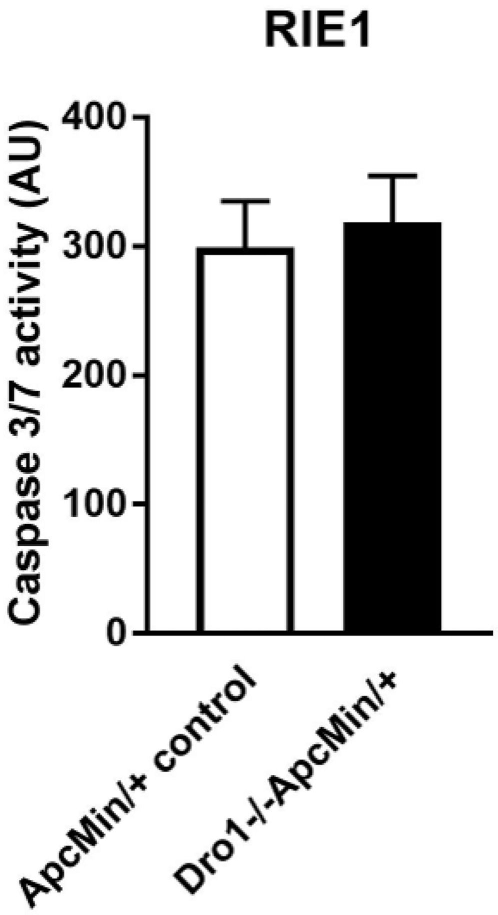

Supplementary Figure 5: Caspase 3/7 activity in RIE1 cells 27 hours after induction of apoptosis by UVB radiation. RIE1 cells were treated with conditioned medium from primary stromal cells generated from tumor-free colon from 5-week-old *Dro1*$^{-/-}$;*Apc*$^{Min/+}$ and *Apc*$^{Min/+}$ control mice (n = 3 biological replicates/genotype). Error bars represent standard error of mean.

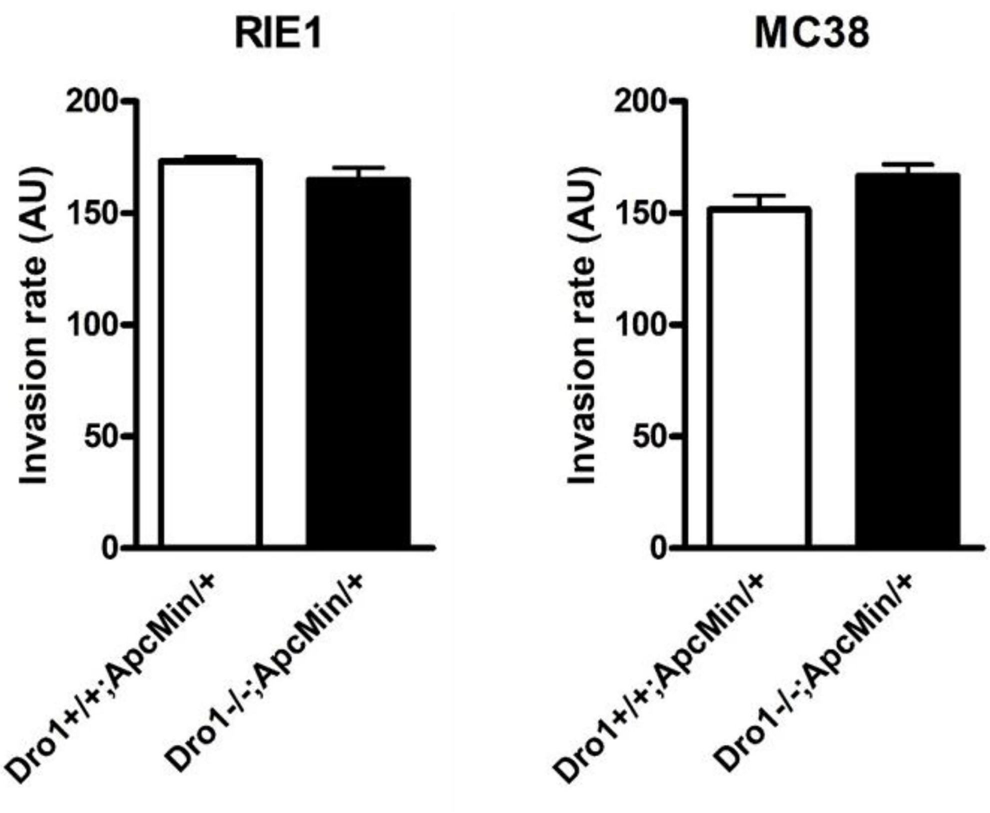

Supplementary Figure 6: Rate of invasion of MC38 colorectal cancer cells and B16 melanoma cells into a basement coated membrane when co-cultivated in a Boyden chamber with primary stromal cells from tumor-free colon of 5-week-old *Dro1*$^{-/-}$;*Apc*$^{Min/+}$ and *Apc*$^{Min/+}$ mice (*n* = 3 biological replicates/genotype). AU, arbitrary units. Error bars represent standard error of mean.

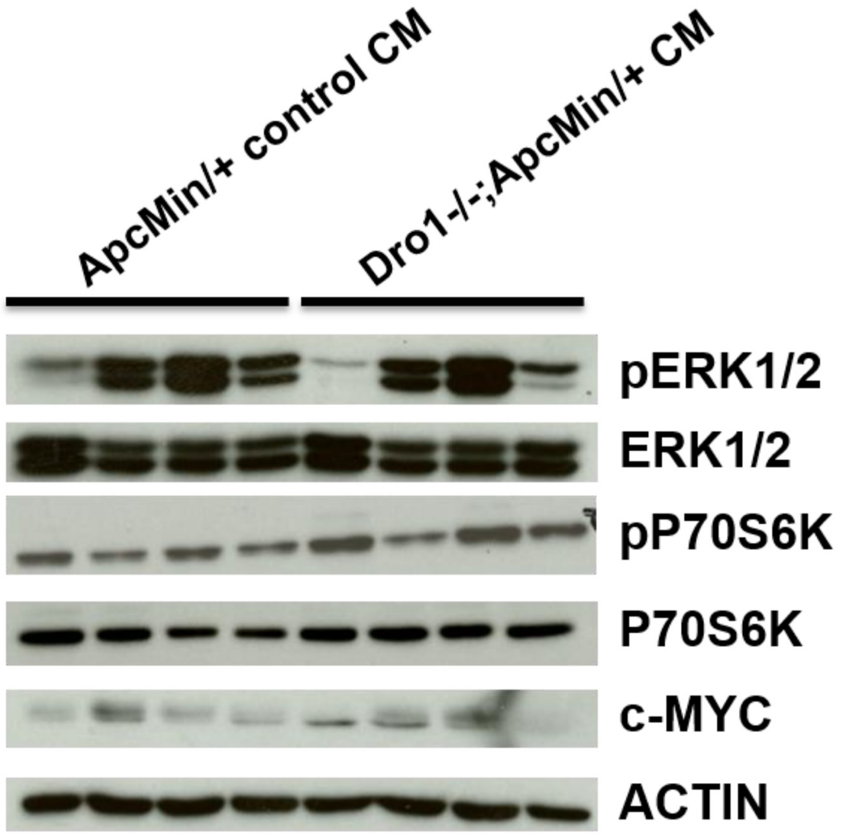

Supplementary Figure 7: Immunoblotting for indicated proteins on total protein extracts from RIE1 cells treated with conditioned medium from *Dro1*$^{-/-}$;*Apc*$^{Min/+}$ and *Apc*$^{Min/+}$ control primary stromal cells for 1 h.

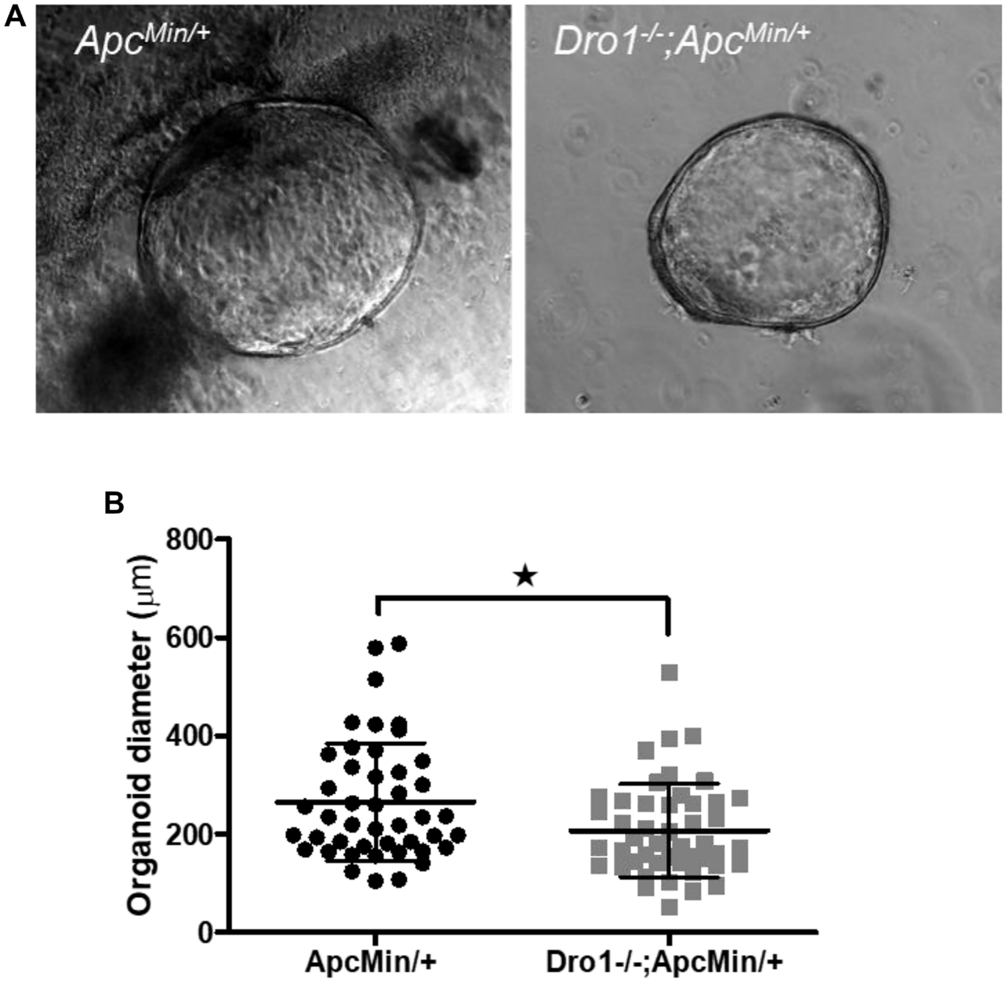

Supplementary Figure 8: (**A**) Morphology and (**B**) diameter of intestinal organoids derived from colon tumors from *Dro1*$^{-/-}$;*Apc*$^{Min/+}$ and *Apc*$^{Min/+}$ control mice. Each dot represents a single organoid, a minimum of 45 organoids was measured for each condition. Error bars represent standard deviations. $^{*}p < 0.05$.

Supplementary Table 1: Histological analysis of colon tumors from moribund *Dro1*$^{fl/fl}$;*Apc*$^{Min/+}$ control and *Dro1*$^{fl/fl}$;*Villin*$^{Cre+}$;*Apc*$^{Min/+}$ mice

	Dro1$^{fl/fl}$;***Apc***$^{Min/+}$	***Dro1***$^{fl/fl}$;***Villin***$^{Cre+}$;***Apc***$^{Min/+}$
Adenoma	24 (88.5%)	17 (94.4%)
Adenocarcinoma	2 (11.5%)	1 (5.6%)
**Total No. of tumors analyzed**	26	18

Supplementary Table 2: Primer sequences for quantitative RT-PCR

Name	Sequence
MouseDro1-FW	5′-CTTCCTCCTGCTCCAGTCAC-3′
MouseDro1-RV	5′-CTGGATAGGCAGTGGTGGTT-3′
HumanDRO1-FW	5′-CCAGAGAAGGAGGAGTGTGC-3′
HumanDRO1-RV	5′-GGGCGAGCTAGTCTCAACAC-3′

Supplementary Table 3: Crypt basal medium (CBM)

Component	Company	Volume
DMEM	Life Technologies	485 ml
100x Pen/Strep	Life Technologies	5 ml
Hepes (1 M)	Life Technologies	5 ml
100x GlutaMax	Life Technologies	5 ml

Supplementary Table 4: Crypt complete medium (CCM)

Component	Company	Volume
CBM	–	38.7 ml
50x B27	Life Technologies	775 µl
100x N2	Life Technologies	387 µl
N-Acetyl-L-cysteine (500 mM)	Sigma	97 µl
Noggin (100 ng/µl)	PeproTech	40 µl
EGF (50 ng/µl)	PeproTech	20 µl
R-Spondin-1 (1 µg/µl)	PeproTech	20 µl
